# Supplementary material for: ZmDRR206 Regulates Nutrient Accumulation in Endosperm through Its Role in Cell Wall Biogenesis during Maize Kernel Development
Source: Int J Mol Sci. 2023 May 13;24(10):8735. doi: 10.3390/ijms24108735 (PMC10218635; doi:10.3390/ijms24108735)
Supplement: Supplementary file 1 [file ijms-24-08735-s001.zip › Supplemental Figures.docx]

**
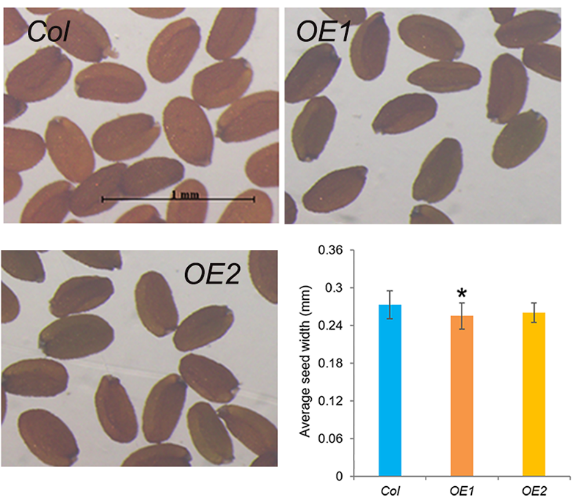
**

**Supplemental Figure 1. *ZmDRR206*-overexpressing induced the transgenic Arabidopsis seeds to be slightly smaller than that of the wild-type (Col).** The comparison of the phenotype of the mature Arabidopsis seeds. Col, the wild-type; *OE1* and *OE2* were two independent *ZmDRR206*-overexpressing transgenic Arabidopsis lines. Left Bottom: The average width of the mature seeds of the Arabidopsis plants. Seeds from the third to eight siliques of the main inflorescence (that turned to be mature earliest) were harvested for the length and width measuring of the seeds.

**
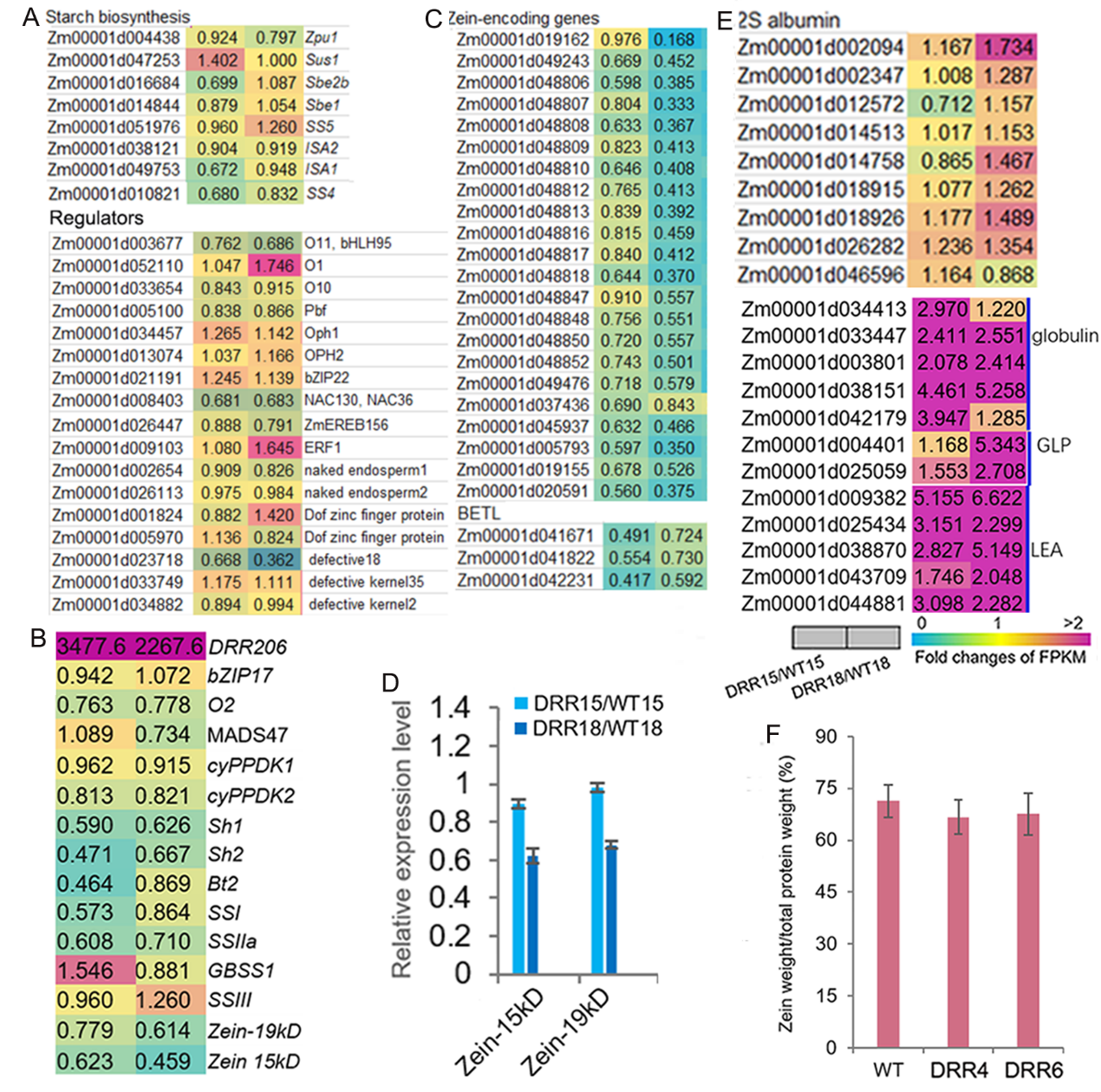
**

**Supplemental Figure 2.** The expression analysis of the kernel-development-regulator and the starch biosynthetic enzyme genes in developing maize kernel. (A-B): Heatmap showing changes in the expression levels of starch biosynthesis- and kernel development-related genes in developing *DRR-OE* relative to that in WT kernel. The relative expression value from transcriptome data of the genes analyzed by qRT-PCR in Figure 5E were listed in (B). (C): Heatmap showing changes in the expression level of *zein* genes in developing *DRR-OE* relative to that in WT kernel. (D): The relative expression level of *Zein-19kD* and *Zein-15kD* in developing *DRR-OE* kernel relative to that in WT kernel by qRT-PCR. (E): Heatmap showing changes in the expression levels of multiple non-zein storage protein genes in developing *DRR-OE* relative to that in WT kernel. The *2S albumin* genes did not differ between the two kernels, while five *globulin* genes, two *germin-like protein* (*GLP*) genes and five *late-embryogenesis protein* (*LEA*) genes were upregulated in developing *DRR-OE* relative to that in WT kernel. WT15/WT18 is the wild-type inbred line LH244 kernel at 15-DAP/18-DAP, DRR15/DRR18 is the *DRR-OE* kernel at 15-DAP/18-DAP. The numerical values are the ratios of the DEGs in the comparison (DRR15/WT15, DRR18/WT18), the background color showed the relative gene expression levels in the comparison (red represents up-regulated, blue is down-regulated, and yellow is no significant change). (F): The zein protein percentage analysis in *DRR-OE* and WT kernel. WT is the wild-type LH244, *DRR4* and *DRR6* are *ZmDRR206* over-expressing transgenic everts *DRR-OE4* and *DRR-OE6*.
